# Subtyping glioblastoma by combining miRNA and mRNA expression data using compressed sensing-based approach

**DOI:** 10.1186/1687-4153-2013-2

**Published:** 2013-01-14

**Authors:** Wenlong Tang, Junbo Duan, Ji-Gang Zhang, Yu-Ping Wang

**Affiliations:** 1 Department of Biomedical Engineering, Tulane University, New Orleans, LA, USA; 2 Department of Biostatistics and Bioinformatics, Tulane University, New Orleans, LA, USA; 3 Center for Systems Biomedicine, Shanghai University for Science and Technology, Shanghai, China

**Keywords:** Glioblastoma, Data integration, Compressed sensing, Classification, mRNA, miRNA

## Abstract

In the clinical practice, many diseases such as glioblastoma, leukemia, diabetes, and prostates have multiple subtypes*.* Classifying subtypes accurately using genomic data will provide individualized treatments to target-specific disease subtypes. However, it is often difficult to obtain satisfactory classification accuracy using only one type of data, because the subtypes of a disease can exhibit similar patterns in one data type. Fortunately, multiple types of genomic data are often available due to the rapid development of genomic techniques. This raises the question on whether the classification performance can significantly be improved by combining multiple types of genomic data. In this article, we classified four subtypes of glioblastoma multiforme (GBM) with multiple types of genome-wide data (e.g., mRNA and miRNA expression) from The Cancer Genome Atlas (TCGA) project. We proposed a multi-class compressed sensing-based detector (MCSD) for this study. The MCSD was trained with data from TCGA and then applied to subtype GBM patients using an independent testing data. We performed the classification on the same patient subjects with three data types, i.e., miRNA expression data, mRNA (or gene expression) data, and their combinations. The classification accuracy is 69.1% with the miRNA expression data, 52.7% with mRNA expression data, and 90.9% with the combination of both mRNA and miRNA expression data. In addition, some biomarkers identified by the integrated approaches have been confirmed with results from the published literatures. These results indicate that the combined analysis can significantly improve the accuracy of classifying GBM subtypes and identify potential biomarkers for disease diagnosis.

## Introduction

Many diseases including cancers have multiple subtypes. For example, leukemia has four main categories: acute lymphoblastic leukemia (ALL), acute myelogenous leukemia, chronic lymphocytic leukemia, and chronic myelogenous leukemia. Each of these categories can be further divided into different subtypes [1]; for example, ALL can be further subtyped into six types [2]. Glioma has four subtypes, including oligodendroglioma, anaplastic oligodendroglioma, anaplastic astrocytoma, and glioblastoma multiforme (GBM) [3]. Prostate cancer has three major subtypes [4]. An accurate and effective classification of those subtypes based on genomic data will result in personalized treatments of the cancer in terms of a particular subtype. In this article, we are interested in the subtyping of GBM, which is a kind of glioma and is the most common form of malignant brain cancer in adults [5]. There is an increasing interest in classifying multiple subtypes of GBM based on its genomic measurements. Most of the existing works are based on gene expression data only. Benjamin et al. [6] classified two types of GBM in adults and found that the genes EGFR and TP53 were important in discriminating the two subtypes. Nutt et al. [7] built a *k*-nearest neighbor model with 20 features to classify 28 glioblastomas and 22 anaplastic oligodendrogliomas and found that the class distinctions were significantly associated with survival outcome (*p* = 0.05). Noushmehr et al. [8] separated a subset of samples in GBM from The Cancer Genome Atlas (TCGA) project, which displayed concerted hypermethylation at a large number of loci. The datasets we used to subtype GBM are also from TCGA. The subtypes of GBM samples in TCGA includes: pro-neural, neural, classical, and mesenchymal [9]. The GBM data we have tested include both miRNA expression and mRNA expression data. The miRNAs, also called microRNAs, are short non-coding RNA molecules that were recently found in all eukaryotic cells except fungi, algae, and marine plants. The human genome may contain over 1,000 miRNAs [10]. Aberrant expressions of miRNAs have been found to be related to many diseases, including cancers [11,12]. They play an essential role in tissue differentiation during normal development and tumorigenesis [13].

In the last decade, the development of genomic techniques enables the availability of multiple data types on the same patient, such as mRNA or gene expression, SNP, miRNA expression, and copy number variation data. It is well recognized that a more comprehensive analysis result could be obtained based on integrating multiple types of genomic data than using an individual dataset. Soneson et al. [14] investigated the correlation between gene expression and copy number alterations using canonical correlation analysis for leukemia data. A web-based platform, called Magellan, was developed for the integrated analysis of DNA copy number and expression data in ovarian cancer [15], which found significant correlation between gene expression and patient survival. Troyanskaya et al. [16] developed a Bayesian framework to combine heterogeneous data sources to predict gene function with improved accuracy. A kernel-based statistical learning algorithm was also proposed in the combined analysis of multiple genome-wide datasets [17]. In this article, we propose a novel classifier based on the compressed sensing (CS) theory that we have been working with.

The CS technique enables compact storage and rapid transmission of large amounts of information. The technique can be used to extract significant statistical information from high-dimensional datasets [18]. The CS technology has been proven to be a powerful tool in the signal processing and statistics fields. It demonstrates that a compressible signal can be recovered from far fewer samples than that needed by the Nyquist sampling theorem [19]. Our recent work used a CS-based detector (CSD) for subtyping leukemia with gene expression data [20]. The CSD achieved high classification accuracies, with 97.4% evaluated with cross-validation and 94.3% evaluated with an independent dataset. The CSD showed better performance in subtyping two types of leukemia compared to some traditional classifiers such as the support vector machine (SVM), indicating the advantage of the CSD in analyzing high-dimensional genomic data. In this article, we extended the CSD to multiple data types and proposed a detector called MCSD. In particular, we applied the MCSD to the subtyping of four types of GBM by combining miRNA expression and mRNA expression data. We present a novel combined analysis method based on the CS and demonstrate that the classification performance can significantly be improved in subtyping four types of GBM, with both miRNA expression and mRNA expression data.

## Methods and materials

### Data collection

The GBM data used in this study are publicly available from the website of TCGA [21]. The patients in the dataset can be classified into four subtypes, i.e., pro-neural, neural, classical, and mesenchymal [9]. The genomic data include miRNA expression (1,510 probes) and mRNA expression data (22,277 probes). We randomly divided the data (including 115 patients with both miRNA and mRNA expression data) into two sets: training and testing datasets. The total number of patients in the training dataset was 60 with 15 patients in each group. The testing dataset had 55 patients, with 17 pro-neural, 3 neural, 17 classical, and 18 mesenchymal subtypes (as listed in Table 1). The same number of patients in each subtype for training data was used for reducing the bias in the model building. Meanwhile, the numbers of patients in training and testing were approximately the same.

**Table 1:** GBM subtypes and their corresponding samples used for the training and the testing

**Glioblastoma subtypes**	**Training (total 60)**	**Testing (total 55)**
Pro-neural	15	17
Neural	15	3
Classical	15	17
Mesenchymal	15	18

These datasets are publically available from the TCGA project.

For multiple types of genomic data (e.g., miRNA expression data, mRNA expression data, etc.), we used ***x***_1*i*_ to denote the data vector for the *i*th sample in data 1 (e.g., miRNA expression), ***x***_2*i*_ to denote the data vector for the *i*th sample in data 2 (e.g., mRNA expression), and ***x***_*ni*_ to denote the data vector for the *i*th sample in data *n*. The combined data for the *i*th sample is $x_{i}={\left(x_{1}\begin{array}{c} T\\ i \end{array},x_{2}\begin{array}{c} T\\ i \end{array},\ldots ,x_{n}\begin{array}{c} T\\ i \end{array}\right)}^{T}$, which is arranged in a cascaded manner.

### MCSD

#### Bayesian classifier

**Figure 1:**
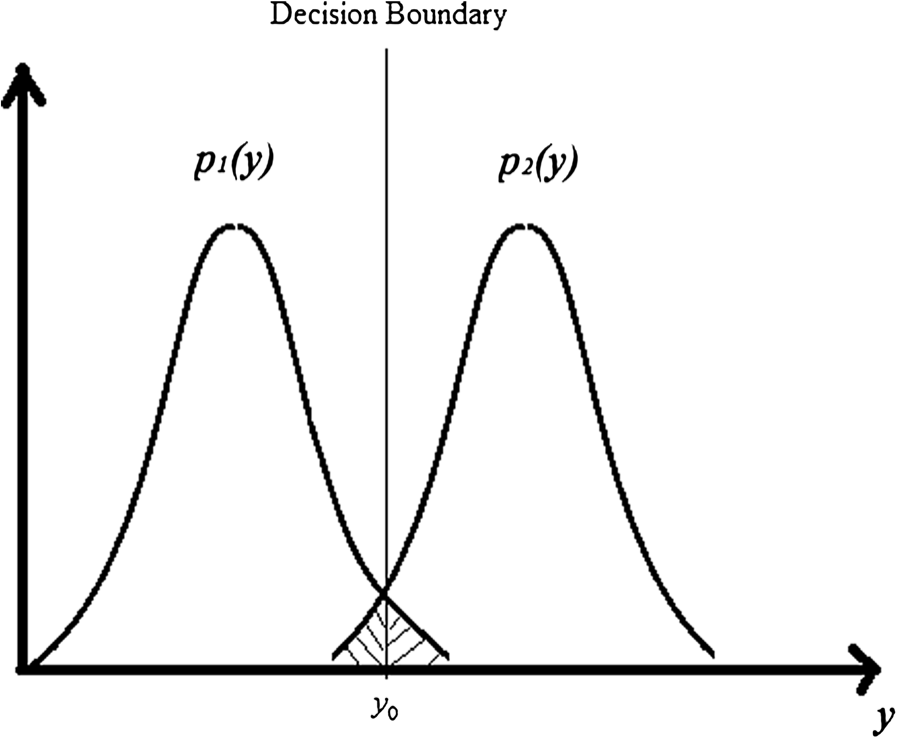
Each probability density function is one-dimensional normal distribution (area under each curve sums to 1).

To classify a given observation ***y*** to one of *n* classes, we define the actual class (“ground truth”) to which it belongs as *g*; the class to which it is assigned (“decision”) as *d*. The *n* classes are defined as: *π*_1,_*π*_2_,…,*π*_*n*_. Let *U*_*πi*_ (***y****,g*) be the utility of assigning ***y***, actually from *π*_***g***_, to *π*_***i***_. The “utility” is negative relevant to the Bayes Risk (BR) [22], which is the minimum classification error. Thus, we make: *U =* 1-BR. The two-class one-dimensional BR (shaded area in Figure 1) can be calculated by

(1) $$\mathrm{BR}=P_{2}\int_{-\infty }^{y_{0}}p_{2}\left(y\right)dy+P_{1}\int_{y_{0}}^{\infty }p_{1}\left(y\right)dy\text{,}$$

where *y*_0_ is the decision boundary, *P*_1_*P*_2_ are the prior probabilities and *P*_1_(*y*),*P*_2_(*y*) are the conditional probability density functions of the two classes, respectively (shown in Figure 1).

Let us extend the BR to *n* classes and *N* dimensions. Then Equation (1) can be rewritten as

(2) $${\mathrm{BR}}_{N}=\sum_{i=1}^{n}\sum_{\begin{array}{l} j=1\\ j\neq i \end{array}}^{n}\;P_{j}\;\int_{\Omega_{i}}p_{j}\left(y\right)\mathrm{dy}\text{,}$$

where *P*_*j*_ is the prior probability of a given subject belonging to the class *π*_*j*_, *j* = 1,…,*n*; *P*_*j*_(*y*) is the conditional probability density function of the class *π*_*j*_, and *Ω*_*i*_ is the Bayesian decision region for class *π*_*i*_[23].

For multi-class classification, an ideal detector should yield

$$P\left(d=\pi_{i}|g=\pi_{j}\right)=\delta_{\mathrm{ij}}\text{,}$$

where

(3) $$\delta_{\mathrm{ij}}=\{\begin{array}{c} 0,\;\\ 1,\; \end{array}\begin{array}{c} \;i\neq j\\ \;i=j \end{array}\text{,}$$

where *P*(*d* = *π*_*i*_|*g* = *π*_*j*_) denotes the probability of assigning a given observation ***y***, actually belonging to *π*_*j*_, to *π*_*i.*_*δ*_*ij*_ is the Kronecker’s delta.

According to the ideal observer decision theory [22], a decision is selected only if its expected utility is greater than the expected utility of any others. Thus, for any given observation ***y***, we decide *d = π*_*i*_ iff

(4) $$E\left\{U_{\pi_{i}}\left(y,g\right)|y\right\}>E\left\{U_{\pi_{j}}\left(y,g\right)|y\right\},i\neq j\;j=1,2,\ldots ,i-1,i+1,\ldots ,n\text{.}$$

From Equation (2) and the relationship of utility and BR, we know “utility” is a number that can be calculated. We denote that number as *U*_*i*|*j*_ to express the utility of assigning a given observation ***y***, actually belonging to *π*_*j*_, to *π*_*i*_. The inequality (4) can be written as

(5) $$\begin{array}{c} \;\overset{n}{\underset{k=1}{\sum }}U_{i|k}P\left(g=\pi_{k}|y\right)>\overset{n}{\underset{k=1}{\sum }}U_{j|k}P\left(g=\pi_{k}|y\right),\\ i\neq j,j=1,2,\ldots ,i-1,i+1,\ldots ,n,\text{.} \end{array}$$

We apply Bayes’ rule

(6) $$P\left(g=\pi_{k}|y\right)=\frac{P_{y}\left(y|g=\pi_{k}\right)P\left(g=\pi_{k}\right)}{P_{y}\left(y\right)}\text{,}$$

where *P*_*y*_(***y***|*g* = *π*_*k*_), *k* = 1,…,*n*, is the probability density function for the signal observations. According to Inequality (5), we decide *d = π*_*i*_ iff

(7) $$\begin{array}{c} \sum_{k=1}^{n}U_{i|k}P\left(g=\pi_{k}\right)p_{y}\left(y|g=\pi_{k}\right)>\sum_{k=1}^{n}U_{j|k}P\left(g=\pi_{k}\right)\\ \;p_{y}\left(y|g=\pi_{k}\right)\;i\neq j\;j=1,2,\ldots ,i-1,i+1,\ldots ,n\text{.} \end{array}$$

That is known as maximum likelihood estimation. Specifically, the class label of the testing sample ***y*** is given by

(8) $$\mathrm{ID}=\underset{l}{\mathrm{argmax}}\left[\sum_{k=1}^{n}U_{l|k}P\left(g=\pi_{k}\right)p_{y}\left(y|g=\pi_{k}\right)\right]\text{.}$$

If we assume *π*_1_, *π*_2_,…,*π*_*n*_ have the same prior probability, i.e., $P\left(g=\pi_{k}\right)=\frac{1}{n}$. The detector (8) can be rewritten as

(9) $$\mathrm{ID}=\underset{l}{\mathrm{argmax}}\left[\sum_{k=1}^{n}U_{l|k}p_{y}\left(y|g=\pi_{k}\right)\right]\text{.}$$

The calculation of the utility is shown in the Additional file 1.

### Dimension reduction using CS

To reduce the dimension of original sample, we design a projection (sparse) matrix ***Φ****,* called compress matrix*.* The generation of the compress matrix can be formulated as a sparse representation problem as in Equation (10)

(10) Y=ΦS,

where $Y=\left\{y_{i}\right\}\in R^{M\times c}$ is the projected sample, *M* is the dimension of the sample after the projection, ***y***_*i*_ is the *i*th column in the compressed signal, *c* is the total number of columns in the compressed signal, $S=\left\{s_{i}\right\}\in R^{N\times c}$ is the original signal, and *N* is the dimension of the original signal and *N >> M*. The matrix $\Phi \in R^{M\times N}$ is a sparse matrix, with most of the entries ‘0’s. The compress matrix ***Φ*** projects the original sample ***S*** to a much smaller dimensional signal ***Y***. The original sample may contain redundancy; through this projection, the original sample can significantly be compressed and compactly represented, which usually lead to better classification performance. Suppose we have *n* groups, with *c*_1_ training samples in group 1, *c*_2_ training samples in group 2, and so forth, *c*_*n*_ training samples in group *n*, and *c* = *c*_1_ + *c*_2_ + ··· + *c*_*n*_ for $S=\left[s_{1,}s_{2},\ldots ,s_{c}\right]\in R^{N\times c}$ and $Y=\left[y_{1,}y_{2},\ldots ,y_{c}\right]\in R^{M\times c}$. The transpose of Equation (10) is

(11) $$S^{T}\Phi^{T}=Y^{T}\text{.}$$

Let ${\left(\Phi^{T}\right)}_{j}\in R^{N\times 1}$ denote the *j*th column of ***Φ***^*T*^, and ${\left(Y^{T}\right)}_{j}\in R^{c\times 1}$ denote the *j*th column of ***Y***^*T*^, where *j* = 1, 2,…,*M*. Then Equation (11) can be rewritten as

(12) $$S^{T}{\left(\Phi^{T}\right)}_{j}={\left(Y^{T}\right)}_{j}\text{.}$$

The linear system given by (12) is an underdetermined system, which can be solved by using *l*-1 norm minimization algorithm such as Homotopy method, or the least angle regression method [24]. The *l*-1 norm optimization problem reads

(13) $${\left(\Phi^{T}\right)}_{j}=\underset{{\left(\Phi^{T}\right)}_{j}}{\mathrm{argmin}}{\left\|{\left(\Phi^{T}\right)}_{j}\right\|}_{1}\text{,}$$

subject to

$$S^{T}{\left(\Phi^{T}\right)}_{j}={\left(Y^{T}\right)}_{j\text{,}}$$

where ‖(***Φ***^*T*^)_*j*_‖_1_ is the *l*-1 norm of the vector (***Φ***^*T*^)_*j*_, i.e., the sum of the absolute values of entries in vector (***Φ***^*T*^)_*j*_. Obviously, the compress matrix ***Φ*** projects the original signal $s_{i}\in R^{N\times 1}$ to a much smaller dimensional signal $\Phi s_{i}\in R^{M\times 1}$. Instead of dealing with the original signal, we only use $\Phi s_{i}\in R^{M\times 1}$ and $\Phi \Phi^{T}\in R^{M\times M}$ in the subtyping procedure, leading to a fast classification.

### Determination of feature vector

We need to select significant features to represent the original data before we classify the data. For each sample, we extracted five feature characteristics [20]: the mean and the standard deviation of each group’s standard deviation (*MeanStd, StdStd*), the standard deviation of the means of all the groups (*StdMean*), and the mean and standard deviation of Pearson’s linear correlation coefficient (*MeanCorr, StdCorr*) between the samples and their class label vector. Therefore, for the *i*th sample, we have a five-dimensional feature vector as follows:

$$V_{i}=\left\{\mathrm{MeanSt}d_{i},\mathrm{StdSt}d_{i},\mathrm{StdMea}n_{i},\mathrm{MeanCor}r_{i}\mathrm{StdCor}r_{i}\right\}$$

where *i* = 1,2,…,*N*, and *N* is the number of samples. Each element in the vector ***V***_*i*_ has been normalized by its overall maximum value so that its value is between 0 and 1, i.e., ***V***_*i*_ ∈ [0, 1]. A number of *M* informative features were selected by setting the threshold values of ***V***_*i*_. If a feature is informative or significant, we expect that the values from different patients within the same subtype are similar while the differences among different subtypes are relatively large. In addition, it is easy to understand that, if the correlation between the feature vector and the class label is high, the feature vector can serve as a significant biomarker to distinguish the subtypes. According to the above analysis, matrix ***Y*** in Equation (10) is built by those features with low *MeanStd, StdStd, StdCorr* while high *StdMean, MeanCorr*, which are significant for the classification.

### Classifier based on CS

In this particular study of subtyping four types of GBM with miRNA expression and mRNA expression data, we make a hypothesis that the data follow a normal distribution. In other words, the probability density function for the data is

(14) $$p_{y}\left(\hat{y}|g=\pi_{k}\right)={\left(2\pi \sigma^{2}\right)}^{-\frac{N}{2}}\exp\left(-\frac{\left\|\hat{y}-s\right\|\begin{array}{c} 2\\ 2 \end{array}}{2\sigma^{2}}\right)\text{,}$$

where $\hat{y}\in R^{N}$ is a given observation; $s\in R^{N}$ is the mean of a sample; and σ is the standard deviation of the data.

After compressing the original sample, the probability density function (Equation 14) is still Gaussian but with different mean and standard deviation given by [18]

(15) $$p_{y}\left(y|g=\pi_{k}\right)=\frac{\exp\left(-\frac{1}{2}{\left(y-\mathrm{\Phi s}\right)}^{T}{\left(\sigma^{2}\Phi \Phi^{T}\right)}^{-1}\left(y-\mathrm{\Phi s}\right)\right)}{{\left|\sigma^{2}\Phi \Phi^{T}\right|}^{\frac{1}{2}}{\left(2\pi \right)}^{\frac{N}{2}}}$$

where $\hat{y}\in R^{M}$ is a compressed observation; $s\in R^{N}$ is a known signal and ***Φ*** is the compress matrix. The MCSD used in this study is constructed by substituting Equation (15) into Equation (9) for maximum likelihood estimation.

The classification algorithm is described as below.

1. Inputs: training dataset and testing dataset

2. Normalize the rows of the training and the testing datasets to the range of [0,1]

3. Select informative features according to the feature selection criteria

4. Calculate compress matrix $\Phi \in R^{M\times N}$ by the training dataset by Equation (14)

5. Identify the class of the compressed testing data by Equation (9), where the probability density function is given by Equation (15).

There are many other classifiers such as SVM that can be used. But our purpose here is to show that dimension reduction with the CS can improve subsequent classification and the often used Bayesian classifier is chosen.

## Results

We subtyped four types of GBM with multiple genomic data types (e.g., miRNA expression, mRNA expression, and their combinations) from TCGA. The MCSD was first trained by the training data with known class labels, and was then employed to detect subtypes in another independent testing dataset. The classification accuracy by the MCSD was compared with that without using MCSD. The classification performance between using the combined data types and using a single type of data was also compared.

Table 2 shows the comparison of the GBM classification accuracy for the testing dataset, with and without the compress matrix used in our algorithm (see Section “Methods and materials”). The results were obtained on three types of data, i.e., miRNA expression data, mRNA expression data, and their combinations. The classification accuracy is defined as the ratio between the number of correctly labeled samples and the number of total samples. The result calculated by the non-compressed detector had a classification accuracy of 41.8% with miRNA expression data. However, when we used the MCSD to classify the four subtypes, the accuracy of classifying the testing dataset was 69.1%, with 54 selected informative features out of 1,510 features. When we tested the classifiers on the mRNA expression data, the result calculated by the non-compressed detector was 32.7%. However, the classification result with the MCSD was 52.7%, which employed a subset of the features, 432 out of 22,277 features.

**Table 2:** Comparison of classification accuracy between MCSD and non-compressed detector using combined and single data type

	**MCSD**		**Non-compressed accuracy (%)**
	**Accuracy (%)**	**Number of features**	
Combined analysis	90.9	121	32.7
miRNA	69.1	54	41.8
Gene expression	52.7	432	32.7

We also tested if the classification performance of the MCSD was better than non-compressed detector in the combined analysis of both miRNA expression and mRNA expression data as shown in Table 2. The subtyping accuracy by the non-compressed detector was 32.7%. The classification accuracy by the MCSD showed a significant improvement over the non-compressed detector. The accuracy was 90.9% (121 informative features selected or 145 informative features selected). The 121 features selected are shown in Additional file 2 with the probes and the corresponding symbols.

**Figure 2:**
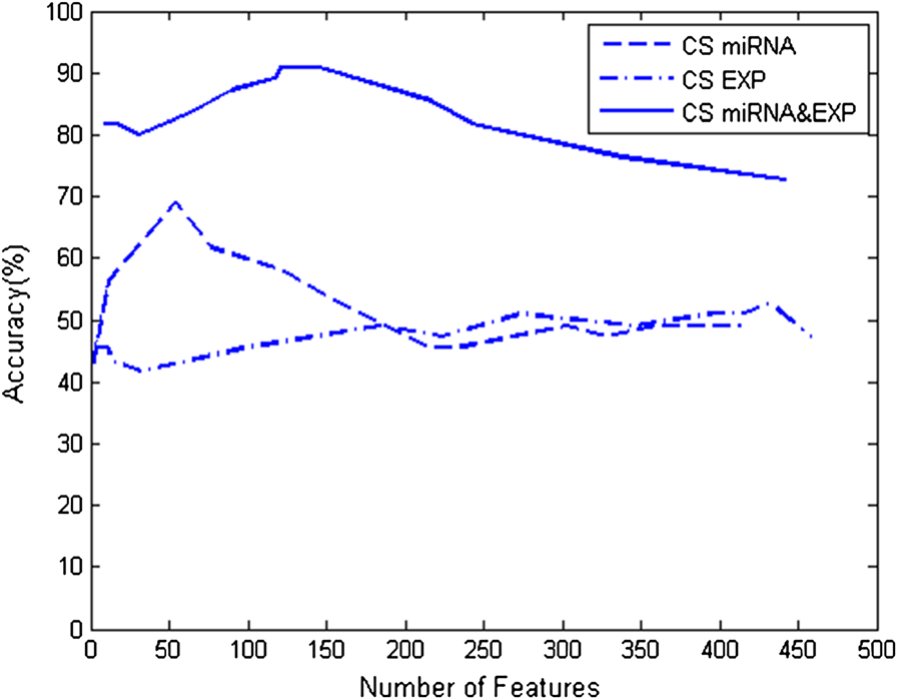
**The comparison of the classification accuracies between the combined analysis and the single data type analysis.** All of them employed MCSD method to subtype four types of GBM. Note that a significant improvement of the classification accuracy has been achieved by using the combined analysis.

Figure 2 demonstrates the classification accuracy when different numbers of informative features were employed. The combined analysis of the two types of genome-wide data was always able to achieve a significant higher subtyping accuracy than any single data type analysis when the same number of informative features were used (with a subset of features less than 450), indicating the advantages of the combined analysis. Figure 2 also shows that the classification accuracy was low when only a few features were used, indicating that the subset was too small to represent the characteristics of the entire dataset. When we increased the number of features used in the MCSD, the classification accuracy went up. The accuracy of classifying the testing dataset reached the highest value, 69.1, 52.7, and 90.9% on the miRNA expression, mRNA expression, and their combinations, respectively. However, more features may also add redundancy and thus cause the decrease of the classification accuracy. Therefore, we conclude that the use of fewer but significant features will achieve better classification accuracy.

**Figure 3:**
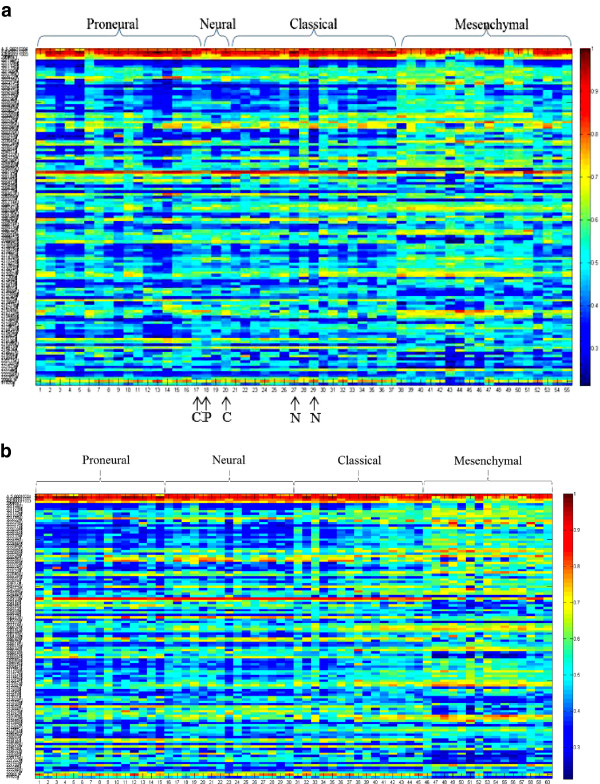
**Display of the selected features in distinguishing the four subtypes of GBM, i.e., pro-neural (P), neural (N), classical (C), and mesenchymal (M) for the testing dataset (a) and the training dataset (b).** 121 features (3 miRNA expression probes on the top and followed by 118 mRNA expression probes) were chosen from both miRNA expression and mRNA expression data. Each row represents a feature and each column represents a sample/patient. Each feature is normalized by the largest value in each row. The samples with arrows were misclassified to the subtypes as denoted by the arrow.

Figure 3a displays the normalized levels of the 121 selected features (118 mRNAs and 3 miRNAs) from both miRNA expression and mRNA expression data for the combined analysis, with the highest classification accuracy of 90.9%. If using the mRNA and miRNA data separately, they only give the accuracy as 49.1 and 47.3%, respectively. The samples with arrows were misclassified to the subtypes pointed by the arrows (e.g., the 17th sample that belongs to pro-neural was misclassified to classical). Each column represents a patient/sample and each row represents a feature (a probe from miRNA expression or mRNA expression data). The four subtypes of GBM are pro-neural, neural, classical, and mesenchymal. Each feature was normalized by the largest value in each row. It can be found that the misclassification only happens among the subtypes of pro-neural, neural, and classical. The number of misclassified samples in each subtype is one sample in pro-neural, two samples in neural, two samples in classical, and zero samples in mesenchymal. The expression levels in the subtype mesenchymal exhibit a significant difference from other three subtypes as shown in Figure 3a. Figure 3b displays the same selected features in the training dataset.

## Conclusion and discussion

In this study, we applied the proposed MCSD to subtype four types of GBM: pro-neural, neural, classical, and mesenchymal with multiple genetic data from TCGA. High classification accuracy was achieved by using CS-based technique (i.e., MCSD) along with the combination of multiple datasets. The results from combining two types of genomic data were compared with those from single type of data. Moreover, the performance of the classification with and without MCSD technique had also been compared. The comparisons showed that the CS-based combined analysis of multiple types of genetic data could significantly improve the accuracy of detecting GBM subtypes.

Combining different types of genomic data allows us to interpret the information in the datasets comprehensively. The information from miRNA and mRNA are complementary to each other; so a combined analysis can give a better result than single data type analysis. miRNAs are a recently discovered class of small non-coding RNAs that regulate gene expression [25], which can be combined with mRNA data for better disease subtyping. However, if no dimension reduction with CS was applied, we found from Table 2 that the classification accuracy from combined analysis was comparable to that from the single mRNA expression because of the redundancy added. The classification performance was significantly improved after we used CS method, indicating that CS may reduce redundancy [26] in the combined datasets and thus improve the classification accuracy.

Informative features/biomarkers selected in this study have also been validated to be associated with GBM and have been reported in the literatures. In the combined data analysis, the 121 features/probes selected (shown in Additional file 2), the 3 miRNA expression probes and 118 mRNA expression probes are listed. Two of the selected miRNAs probes that represent the same miRNA, “hsa-miR-9” (sequence “TCATACAGCTAGATAACCAA”), have been validated to have stemness potential and chemoresistance to GBM cells [27-29], and known to be specifically expressed during brain neurogenesis. In the listed mRNA expression probes, the four probes of “CD44” and the three probes of “ASCL1” are selected. Both of the genes have been validated as biomarkers in subtyping GBM in multiple genomic studies [9,30-32]. It demonstrates the significance of “CD44” and “ASCL1” in discriminating different subtypes of GBM. The three probes from “THBS1” are also selected in the 121 probes list. “THBS1” is a subunit of a disulfide-linked homotrimeric protein. This protein has been shown to play roles in platelet aggregation, angiogenesis, and tumorigenesis [33]. “THBS1” is also a major activator of “TGFB1” and the “TGFB1” expression is associated with GBM [34]. Moreover, it has been found that “TbRII”, a receptor of “TGFB1”, has a strong relationship with human malignant glioblastoma cells [35]. There are biomarkers listed in Additional file 2 that have not been reported yet. However, they may be potential biomarkers for GBM, deserving further study.

We also performed Gene Ontology (GO) analyses to determine that these genes were enriched in specific GO terms (biological processes). The GO term “antigen processing” and presentation “lymphocyte mediated immunity” (*p* = 1.78 × 10^–6^), and several GO terms related to wounding healing [e.g. “response to wounding” (*p* = 1.26 × 10^–8^); “wound healing” (*p* = 2.44 × 10^–6^)], and cell adhesion [e.g. “biological adhesion” (*p* = 6.53 × 10^–7^); “cell adhesion” (*p* = 6.41 × 10^–7^)] showed highly significant enrichment for our selected genes. These results were expected. Taking “lymphocyte mediated immunity”-related GO categories as an example, lymphocyte-mediated cellular responses play a critical role in the body’s ability to generate an antitumor immune response, and activation status of lymphocytes is an important determinant of sensitivity to tumor-mediated apoptosis [36]. In addition, according to previous studies, the miRNAs we identified are related to glioblastoma. For example, it was found that “has-miR-9” inhibit differentiation of glioblastoma stem cells, and the calmodulin-binding transcription activator 1 (CAMTA1) as “has-miR-9” target is a tumor suppressor in glioblastoma [37].

To test the stability of the classification results, the samples in training and testing were randomly rearranged ten more times. The number of samples from each subtype in training and testing was maintained the same as in the description in the section “Data collection”. The overall classification rate has an average value of 87.1% with a standard deviation of 4.5%, indicating that the results are rather robust.

In summary, we have developed a CS-based technique for combining multiple genomic data to subtype glioblastoma more accurately. The biomarkers identified with our approaches have also been validated or reported in some existing literatures, indicating that the integrated approach can provide comprehensive information for better disease diagnosis.

## Competing interests

The authors declare that they have no competing interests.

## Supplementary Material

Additional file 1 **Calculation of *U *for the MCSD.** Click here for file

Additional file 2 List of 121 selected features. Click here for file

## References

[B1] Leukemia-Topic Overviewhttp://www.webmd.com/cancer/tc/leukemia-topic-overview

[B2] Yeung KY Bumgarner RE Raftery AE Bayesian model averaging: development of an improved multi-class, gene selection and classification tool for microarray data Bioinformatics 2005 21 2394 2402 10.1093/bioinformatics/bti319 15713736

[B3] Seungchan K Dougherty ER Shmulevich I Hess KR Hamilton SR Trent JM Fuller GN Zhang W Identification of combination gene sets for glioma classification Mol 2002 1 1229 1236 12479704

[B4] Lapointe J Li C Higgins JP Rijn M Bair E Montgomery K Ferrari M Egevad L Rayford W Bergerheim U Ekman P DeMarzo AM Tibshirani R Botstein D Brown PO Brooks JD Pollack JR Gene expression profiling identifies clinically relevant subtypes of prostate cancer PNAS 2003 101 811 816 14711987 10.1073/pnas.0304146101 PMC321763

[B5] Ohgaki H Kleihues P Epidemiology and etiology of gliomas Acta Neuropathol 2005 109 93 108 10.1007/s00401-005-0991-y 15685439

[B6] Benjamin R Capparella J Brown A Classification of glioblastoma multiforme in adults by molecular genetics Cancer J 2003 9 82 90 10.1097/00130404-200303000-00003 12784873

[B7] Nutt CL Mani DR Betensky RA Tamayo P Cairncross JG Ladd C Pohl U Hartmann C McLaughlin ME Batchelor TT Black PM Deimling AV Pomeroy SL Golub TR Louis DN Gene expression-based classification of malignant gliomas correlates better with survival than histological classification Cancer Res 2003 63 1602 1607 12670911

[B8] Noushmehr H Weisenberger DJ Diefes K Phillips HS Pujara K Berman BP Pan F Pelloski CE Sulman EP Bhat KP Verhaak RGW Hoadley KA Hayes DN Perou CM Schmidt HK Ding L Wilson RK Den Berg DV Shen H Bengtsson H Neuvial P Cope LM Buckley J Herman JG Baylin SB Laird PW Aldape K The cancer genome atlas research network. Identification of a CpG island methylator phenotype that defines a distinct subgroup of glioma Cancer Cell 2010 17 510 522 10.1016/j.ccr.2010.03.017 20399149 PMC2872684

[B9] Verhaak RGW Hoadley KA Purdom E Wang V Qi Y Wilkerson MD Miller CR Ding L Golub T Mesirov JP Alexe G Lawrence M Kelly MO Tamayo P Weir BA Gabriel S Winckler W Gupta S Jakkula L Feiler HS Hodgson JG James CD Sarkaria JN Brennan C Kahn A Spellman PT Wilson RK Speed TP Gray JW Meyerson M Integrated genomic analysis identifies clinically relevant subtypes of Glioblastoma characterized by abnormalities in PDGFRA, IDH1, EGFR, and NF1 Cancer Cell 2010 17 98 110 10.1016/j.ccr.2009.12.020 20129251 PMC2818769

[B10] Bentwich I Avniel A Karov Y Aharonov R Gilad S Barad O Barzilai A Einat P Einav U Meiri E Sharon E Spector Y Bentwich Z Identification of hundreds of conserved and nonconserved human microRNAs Nat 2005 37 766 770 10.1038/ng1590 15965474

[B11] Fasanaro P Greco S Ivan M Capogrossi M Martelli F MicroRNA: emerging therapeutic targets in acute ischemic diseases Pharmacol 2010 125 92 104 10.1016/j.pharmthera.2009.10.003 19896977

[B12] Iorio MV Ferracin M Liu C-G Veronese A Spizzo R Sabbioni S Magri E Pedriali M Fabbri M Campiglio M Ménard S Palazzo JP Rosenberg A Musiani P Volinia S Nenci I Calin GA Querzoli P Negrini M Croce CM MicroRNA gene expression deregulation in human breast cancer Cancer Res 2005 65 7065 7070 10.1158/0008-5472.CAN-05-1783 16103053

[B13] Bishop JA Benjamin H Cholakh H Chajut A Clark DP Westra WH Accurate classification of non-small cell lung carcinoma using a novel MicroRNA-based approach Clin 2010 16 610 619 10.1158/1078-0432.CCR-09-2638 20068099

[B14] Soneson C Lilljebjörn H Fioretos T Fontes M Integrative analysis of gene expression and copy number alterations using canonical correlation analysis BMC Bioinforma 2010 11 191 10.1186/1471-2105-11-191 PMC2873536 20398334

[B15] Kingsley CB Kuo W-L Polikoff D Berchuck A Gray JW Jain AN Magellan: a web based system for the integrated analysis of heterogeneous biological data and annotations; application to DNA copy number and expression data in ovarian cancer Cancer Inf 2006 2 10 21 PMC2675496 19458754

[B16] Troyanskaya OG Dolinski K Owen AB Altman RB Botstein D A Bayesian framework for combining heterogeneous data sources for gene function prediction (in Saccharomyces cerevisiae) PNAS 2003 100 8348 8353 10.1073/pnas.0832373100 12826619 PMC166232

[B17] Lanckriet GRG Bie TD Cristianini N Jordan MI Noble WS A statistical framework for genomic data fusion Bioinformatics 2004 20 2626 2635 10.1093/bioinformatics/bth294 15130933

[B18] Davenport MA Wakin MB Baraniuk RG Detection and estimation with compressive measurements. Technical Report 2007

[B19] Cand`es EJ Wakin MB An introduction to compressive sampling IEEE Signal Process 2008 25 2 21 30

[B20] Tang W Cao H Duan J Wang Y-P A compressed sensing based approach for subtyping of leukemia from gene expression data J 2011 9 631 645 10.1142/S0219720011005689 PMC4159949 21976380

[B21] TCGA Data http://tcga-data.nci.nih.gov/tcga/tcgaHome2.jsp

[B22] Edwards DC Metz CE Kupinski MA Ideal observers and optimal ROC hypersurfaces in N-class classification IEEE Trans 2004 23 891 895 10.1109/TMI.2004.828358 PMC2464283 15250641

[B23] Starks SA Kreinovich V Environmentally-oriented processing of multi-spectral satellite images: new challenges for Bayesian methods Proceedings of the 17th International Workshop on Maximum Entropy and Bayesian Methods of Statistical Analysis, Boise, Idaho 1998 271

[B24] Efron B Hastie T Johnstone I Tibshirani R Least angle regression Ann 2004 32 407 451 10.1214/009053604000000067

[B25] Volinia S Calin GA Liu C-G Ambs S Cimmino A Petrocca F Visone R Iorio M Roldo C Ferracin M Prueitt RL Yanaihara N Lanza G Scarpa A Vecchione A Negrini M Harris CC Croce CM A microRNA expression signature of human solid tumors defines cancer gene targets PNAS 2006 103 2257 2261 10.1073/pnas.0510565103 16461460 PMC1413718

[B26] Tsaig Y Donoho DL Extensions of compressed sensing Signal Process 2006 86 549 571 10.1016/j.sigpro.2005.05.029

[B27] Jeon H-M Sohn Y-W Oh S-Y Kim S-H Beck S Kim S Kim H ID4 imparts chemoresistance and cancer stemness to glioma cells by derepressing miR-9*-mediated suppression of SOX2 Cancer Res 2011 71 3410 3421 10.1158/0008-5472.CAN-10-3340 21531766

[B28] Ko MH Kim S Hwang W Ko HY Kim YH Lee DS Bioimaging of the unbalanced expression of microRNA9 and microRNA9* during the neuronal differentiation of P19 cells FEBS J 2008 275 2605 2616 10.1111/j.1742-4658.2008.06408.x 18410378

[B29] Yoo AS Staahl BT Chen L Crabtree GR MicroRNA-mediated switching of chromatin-remodelling complexes in neural development Nature 2009 460 642 646 19561591 10.1038/nature08139 PMC2921580

[B30] Liang Y Diehn M Watson N Bollen AW Aldape KD Nicholas MK Lamborn KR Berger MS Botstein D Brown PO Israel MA Gene expression profiling reveals molecularly and clinically distinct subtypes of glioblastoma multiforme PNAS 2005 102 5814 5819 10.1073/pnas.0402870102 15827123 PMC556127

[B31] Ariza A López D Mate JL Isamat M Musulen E Pujol M Ley A Navas-palacios J Role of CD44 in the invasiveness of glioblastoma multiforme and the noninvasiveness of meningioma: an immunohistochemistry study Hum 1995 26 1144 1147 10.1016/0046-8177(95)90278-3 7557949

[B32] Phillips HS Kharbanda S Chen R Forrest WF Soriano RH Wu TD Misra A Nigro JM Colman H Soroceanu L Williams PM Modrusan Z Feuerstein BG Aldape K Molecular subclasses of high-grade glioma predict prognosis, delineate a pattern of disease progression, and resemble stages in neurogenesis Cancer Cell 2006 9 157 173 10.1016/j.ccr.2006.02.019 16530701

[B33] Galvez AF Huang L Magbanua MMJ Dawson K Rodriguez RL Differential expression of thrombospondin (THBS1) in tumorigenic and nontumorigenic prostate epithelial cells in response to a chromatin-binding soy peptide Nutr. Cancer 2011 63 623 636 10.1080/01635581.2011.539312 21526452 PMC3210036

[B34] Lin B Madan A Yoon J-G Fang X Yan X Kim T-K Hwang D Hood L Foltz G Massively parallel signature sequencing and bioinformatics analysis identifies up-regulation of TGFBI and SOX4 in human glioblastoma PLoS One 2010 5 e10210 10.1371/journal.pone.0010210 20419098 PMC2856677

[B35] Wesolowska A Sliwa M Kaminska B Development of siRNA against TbRII blocking efficiently TGFb1 signaling pathways in glioma cells Eur 2004 271 35 58 Supplement 1 July: abstract number P2.5-05

[B36] Chahlavi A Rayman P Richmond A-L Biswas K Zhang R Vogelbaum M Tannenbaum C Barnett G Finke J-H Glioblastomas induce T-lymphocyte death by two distinct pathways involving gangliosides and CD70 Cancer Res 2005 65 5428 5438 10.1158/0008-5472.CAN-04-4395 15958592

[B37] Gil-Ranedo J Mendiburu-Elicabe M Garcia-Villanueva M Medina D del Alamo M Izquierdo M An off-target nucleostemin RNAi inhibits growth in human glioblastoma-derived cancer stem cells PLoS One 2011 6 e28753 10.1371/journal.pone.0028753 22174890 PMC3236221

