# Supplementary material for: Subtyping glioblastoma by combining miRNA and mRNA expression data using compressed sensing-based approach
Source: EURASIP J Bioinform Syst Biol. 2013 Jan 14;2013(1):2. doi: 10.1186/1687-4153-2013-2 (PMC3651309; doi:10.1186/1687-4153-2013-2)
Supplement: Additional file 1 — Calculation of U for the MCSD. [file 1687-4153-2013-2-S1.doc]

1. ***Calculation of U for the MCSD***

In the classification work, we compress the original data from *N* dimensions to *M* dimensions by using the sparse transformation matrix$\Phi$. In that case, the Bayes risk is dependent on the matrix$\Phi$. Thus, the Eq. (2) can be written as:

$BR_{M}\left(\Phi\right)=\sum_{i=1}^{n} \sum_{\begin{matrix} j=1 \\ i\neq j \end{matrix}}^{n} P_{j}\int_{\Omega_{i}\left(\Phi\right)} p_{j}\left(y|g=\pi_{j},\Phi\right)dy$, (A1)

where $P_{j}$ is the prior probability of a given subject belonging to the class$\pi_{j}$, and the conditional probability density functions $p_{j}$ are expressed as in Eq. (15), $\Omega_{i}\left(\Phi\right)$ is the Bayesian decision region in *M* dimension space for class $\pi_{i}$. According to the definition of $U_{i|j}$, the utility of assigning a given observation $y$, actually belonging to $\pi_{j}$, to $\pi_{i}$, we define:

$BR_{M}\left(\Phi\right)_{i|j}=P_{j}\int_{\Omega_{i}\left(\Phi\right)} p_{j}\left(y|g=\pi_{j},\Phi\right)dy$. (A2)

So, $U_{i|j}=1-BR_{M}\left(\Phi\right)_{i|j}$.

The integrals in Eq. (A2) can be replaced by their estimates [1]:

$BR_{M}\left(\Phi\right)_{i|j}=P_{j}\frac{1}{N_{j}}\sum_{y_{k}^{j}\in\Omega_{i}\left(\Phi\right)\cap S^{j}} 1$, (A3)

where $S^{j}=\left\{y_{k}^{j}\in E^{m}\left(\Phi\right)|k=1,...,N_{j}\right\}$ is a set of $N_{j}$labeled samples drawn from the class $\pi_{j}$.

Reference

1. RJR De Figueiredo, Optimal linear and nonlinear feature extraction from several Gaussian pattern class, in Proceedings of the Second Joint International Conference on Pattern Recognition, Copenhagen, Denmark, 1974.
