# Supplementary material for: Subtyping glioblastoma by combining miRNA and mRNA expression data using compressed sensing-based approach
Source: EURASIP J Bioinform Syst Biol. 2013 Jan 14;2013(1):2. doi: 10.1186/1687-4153-2013-2 (PMC3651309; doi:10.1186/1687-4153-2013-2)
Supplement: Additional file 2 — List of 121 selected features. [file 1687-4153-2013-2-S2.doc]

***List of 121 selected features***

| Probe ID | Symbol |
| --- | --- |
| A_25_P00010204' | hsa-miR-22 |
| 'A_25_P00011001' | hsa-miR-9 |
| 'A_25_P00011003' | hsa-miR-9 |
| '200884_at' | CKB |
| '201108_s_at' | THBS1 |
| '201109_s_at' | THBS1 |
| '201110_s_at' | THBS1 |
| '201170_s_at' | BHLHE40 |
| '201505_at' | LAMB1 |
| '201798_s_at' | MYOF |
| '202022_at' | ALDOC |
| '202238_s_at' | NNMT |
| '202310_s_at' | COLIA1 |
| '202311_s_at' | COLIA1 |
| '202628_s_at' | SERPINE1 |
| '202637_s_at' | ICAM1 |
| '202638_s_at' | ICAM1 |
| '202733_at' | P4HA2 |
| '202796_at' | SYNPO |
| '202833_s_at' | SERPINA1 |
| '202856_s_at' | SLC16A3 |
| '202948_at' | IL1R1 |
| '202952_s_at' | ADAM12 |
| '202998_s_at' | LOXL2 |
| '203065_s_at' | CAV1 |
| '203234_at' | UPP1 |
| '203240_at' | FCGBP |
| '203295_s_at' | ATP1A2 |
| '203296_s_at' | ATP1A2 |
| '203305_at' | F13A1 |
| '203325_s_at' | COL5A1 |
| '203510_at' | MET |
| '203570_at' | LOXL1 |
| '203645_s_at' | CD163 |
| '203724_s_at' | RUFY3 |
| '203851_at' | IGFBP6 |
| '203854_at' | CFI |
| '204017_at' | KDELR3 |
| '204222_s_at' | GLIPR1 |
| '204273_at' | EDNRB |
| '204298_s_at' | LOX |
| '204490_s_at' | CD44 |
| '204646_at' | DPYD |
| '204685_s_at' | ATP2B2 |
| '205103_at' | C1orf61 |
| '205143_at' | NCAN |
| '205184_at' | GNG4 |
| '205358_at' | GRIA2 |
| '205413_at' | MPPED2 |
| '205499_at' | SRPX2 |
| '205638_at' | BAI3 |
| '205729_at' | OSMR |
| '206453_s_at' | NDRG2 |
| '206584_at' | LY96 |
| '207103_at' | KCND2 |
| '207714_s_at' | SERPINH1 |
| '208637_x_at' | ACTN1 |
| '208747_s_at' | C1S |
| '208789_at' | PTRF |
| '208790_s_at' | PTRF |
| '209156_s_at' | COL6A2 |
| '209292_at' | ID4 |
| '209436_at' | SPON1 |
| '209514_s_at' | RAB27A |
| '209515_s_at' | RAB27A |
| '209732_at' | CLEC2B |
| '209835_x_at' | CD44 |
| '209914_s_at' | NRXN1 |
| '209987_s_at' | ASCL1 |
| '209988_s_at' | ASCL1 |
| '210845_s_at' | PLAUR |
| '210889_s_at' | FCGR2B |
| '210916_s_at' | CD44 |
| '210951_x_at' | RAB27A |
| '211395_x_at' | FCGR2C |
| '211429_s_at' | SERPINA1 |
| '211612_s_at' | IL13RA1 |
| '211651_s_at' | LAMB1 |
| '211864_s_at' | MYOF |
| '212014_x_at' | CD44 |
| '212067_s_at' | C1R |
| '212097_at' | CAV1 |
| '212308_at' | CLASP2 |
| '212488_at' | COL5A1 |
| '212543_at' | AIM1 |
| '213418_at' | HSPA6 |
| '213601_at' | SLIT1 |
| '213609_s_at' | SEZ6L |
| '213768_s_at' | ASCL1 |
| '213790_at' | ADAM12 |
| '213825_at' | OLIG2 |
| '213994_s_at' | SPON1 |
| '214279_s_at' | NDRG2 |
| '214762_at' | ATP6V1G2 |
| '215049_x_at' | CD163 |
| '215223_s_at' | SOD2 |
| '215446_s_at' | LOX |
| '217388_s_at' | KYNU |
| '217562_at' | FAM5C |
| '217966_s_at' | FAM129A |
| '217967_s_at' | FAM129A |
| '218424_s_at' | STEAP3 |
| '218454_at' | PLBD1 |
| '218983_at' | C1RL |
| '219107_at' | BCAN |
| '219196_at' | SCG3 |
| '219386_s_at' | SLAMF8 |
| '219415_at' | TTYH1 |
| '219434_at' | TREM1 |
| '219537_x_at' | DLL3 |
| '219564_at' | KCNJ16 |
| '219945_at' | DDX25 |
| '220115_s_at' | CDH10 |
| '221207_s_at' | NBEA |
| '221623_at' | BCAN |
| '221730_at' | COL5A2 |
| '222288_at' | TRDN |
| '222294_s_at' | CADM4 |
| '222301_at' | C1orf61 |
| '39966_at' | CSPG5 |
| '91920_at' | BCAN |
